# Effects of Integrated Rice-Frog Farming on Paddy Field Greenhouse Gas Emissions

**DOI:** 10.3390/ijerph16111930

**Published:** 2019-05-31

**Authors:** Kaikai Fang, Xiaomei Yi, Wei Dai, Hui Gao, Linkui Cao

**Affiliations:** School of Agriculture and Biology, Shanghai Jiao Tong University, 800 Dongchuan Road, Shanghai 200240, China; fangkaikai@sjtu.edu.cn (K.F.); yixiaomei@sjtu.edu.cn (X.Y.); dw0728@sjtu.edu.cn (W.D.); hgao13@sjtu.edu.cn (H.G.)

**Keywords:** integrated rice-frog farming, fertilization, methane, nitrous oxide, global warming potential, structural equation model

## Abstract

Integrated rice-frog farming (IRFF), as a mode of ecological farming, is fundamental in realizing sustainable development in agriculture. Yet its production of greenhouse gas (GHG) emissions remains unclear. Here, a randomized plot field experiment was performed to study the GHG emissions for various farming systems during the rice growing season. The farming systems included: conventional farming (CF), green integrated rice-frog farming (GIRF), and organic integrated rice-frog farming (OIRF). Results indicate that the cumulative methane (CH_4_) emissions from the whole growth period were divergent for the three farming systems, with OIRF having the highest value and CF having the lowest. For nitrous oxide (N_2_O) emissions, the order is reversed. IRFF significantly increased the dissolved oxygen (DO), soil redox potential (Eh), total organic carbon (TOC) content, and soil C:N ratio, which is closely related to GHG emissions in rice fields. Additionally, the average emissions of carbon dioxide (CO_2_) from soils during rice growing seasons ranged from 2312.27 to 2589.62 kg ha^−1^ and showed no significant difference in the three treatments. Rice yield in the GIRF and OIRF were lower (2.0% and 16.7%) than the control. The CH_4_ emissions contributed to 83.0–96.8% of global warming potential (GWP). Compared to CF, the treatment of GIRF and OIRF increased the GWP by 41.3% and 98.2% during the whole growing period of rice, respectively. IRFF significantly increased greenhouse gas intensity (GHGI, 0.79 kg CO_2_-eq ha^−1^ grain yield), by 91.1% over the control. Compared to the OIRF, GIRF decreased the GHGI by approximately 39.4% (0.59 kg CO_2_-eq ha^−1^ grain yield), which was 44.2% higher than that of the control. The results of structural equation model showed that the contribution of fertilization to CH_4_ emissions in paddy fields was much greater than that of frog activity. Moreover, frog activity could decrease GWP by reducing CH_4_ emissions from rice fields. And while GIRF showed a slight increase in GHG emissions, it could still be considered as a good strategy for providing an environmentally-friendly option in maintaining crop yield in paddy fields.

## 1. Introduction

Rice is the foremost staple food crop for nearly 50% of the current population in the world [1,2,3]. Nevertheless, a recent estimate of cropland GHG emissions indicates that paddy fields account for 48% of the global budget of GHG emissions primarily through discharges of CH_4_ and N_2_O [4]. Globally, rice fields are significant sources of atmospheric CH_4_ and N_2_O, and they are major contributors to global warming [5,6,7]. They exhibit relative GWP of 28 and 265 times that of CO_2_ over a 100-year timescale [8]. Climate change caused by GHG emissions will have a huge impact on agriculture areas. Extreme weather events may result in lower harvestable yields, higher yield variability, and reduction in suitable areas for traditional crops [9]. Moreover, roughly 70% of climate-induced changes in the agricultural output result from variations in frequency and area [10]. Clearly, the impact of climate change on agricultural production is highly consequential. Recently, a comprehensive report has suggested that GHG emissions create a pervasive threat to humanity by intensifying multiple hazards which render people more vulnerable [11]. Therefore, reducing GHG emissions from rice production is a paramount issue requiring immediate action [12].

In the modern agricultural industry, rice yields and environmental impacts have to be evaluated simultaneously when determining the appropriate soil management strategy [13]. More environmentally friendly agricultural systems are being explored that results in higher rice productivity for food security and with lower net GWP and GHGI [14]. For example, the presence of frogs, ducks, and fish can reduce the occurrence of diseases, pests, and weeds in rice fields [15,16]. Long-term rotation of rice-shrimp farming helps improve soil’s physical and chemical properties and improves soil nutrient content [17]. Research aimed at improving quality and increasing efficiency in IRFF have been conducted that minimized the use of pesticides and fertilizers, and have shown positive results. Liu et al. [18] carried out an experiment of raising bullfrogs in paddy fields. The results showed that the application of 900 and 1500 bullfrogs per hectare decreased the planthopper population by 60% to 70% in paddy fields. Compared with the controlled farming, the seed setting rate of IRFF (900 and 1500 bullfrogs ha^−1^) increased by 13.8% and 15.8%, respectively. Non-pollution rice produced by IRFF fills the gap of ecological products in the rice market.

Soil fertilization is an essential factor affecting GHG emissions in rice fields, and it is also the basis for ensuring high yield and good quality of rice [19]. Studies have shown that fertilizer type plays a significant impact on GHG emissions. The application of chemical nitrogen fertilizer leads to increases in N_2_O emissions [20,21]. The reduction of nitrogen fertilizer and the application of slow-release nitrogen fertilizer can significantly reduce the total N_2_O emissions from paddy soils. The application of organic fertilizer can increase TOC content and promote the emissions of CH_4_ in paddy soils [22].

Previous studies have been mainly restricted in analyzing GHG emissions from single farming systems such as rice-duck, rice-fish, and rice-frog, or by the effects of different fertilization levels [23,24]. Few studies have focused on GHG emissions from paddy fields under integrated rice-frog farming. Currently, the combined effects of integrated rice-frog farming and soil fertilization on GHG emissions have not been considered. Here, multiple data processing methods have been adopted to analyze the influence of this conjoined system on GHG emissions in paddy fields. Also, the structural equation model was adopted in the discussion. Structural equation modeling effectively integrates factor analysis, regression analysis, and quantitative analysis. The aims of this study are as follows: (1) to evaluate the impacts of interaction between rice-frog co-cropping and fertilizer on soil GHG emissions in paddy fields, (2) to recommend the best combination of rice field management to reduce GHG emissions while maintaining rice yield, and (3) to provide the scientific basis for ecological rice cultivation.

## 2. Materials and Methods

### 2.1. Experimental Site

**Figure 1:**
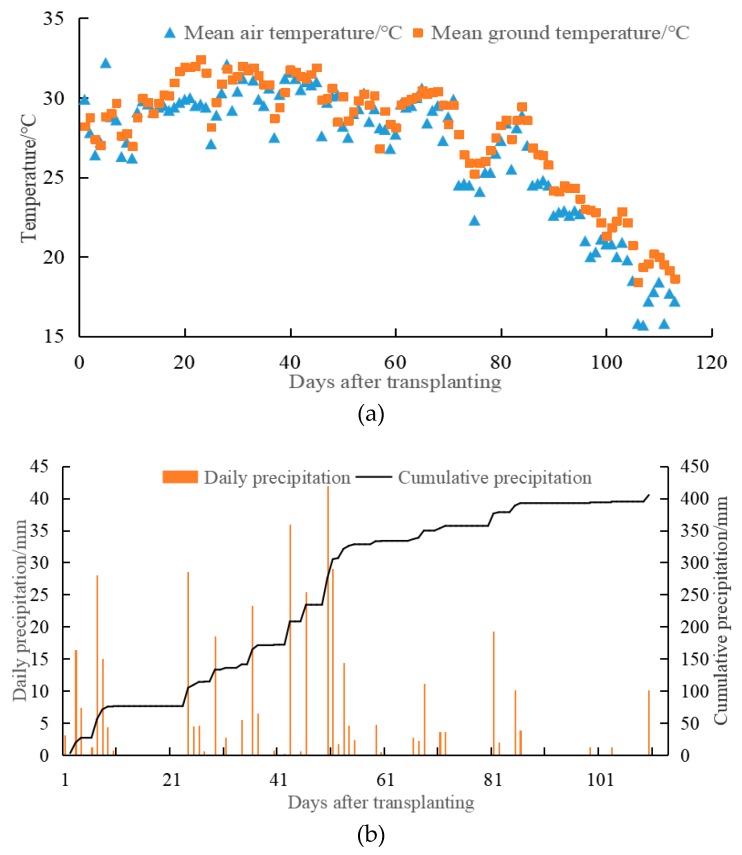
Climate conditions of experimental site during the rice growing seasons in 2018. (**a**): Soil and air temperature; (**b**): Daily and cumulative precipitation.

The field experiment was conducted in a typical rice farm setting at the Modern Agricultural Park of Qingpu, Shanghai, China (121°01′ E, 31°08′ N). Rice (*Oryza sativa* L.) is the dominant crop in the district and is grown once a year [25]. The regional climate is characterized by a subtropical monsoon climate, with an average annual air temperature of 15.5 °C and precipitation of 1056 mm. The average annual sunshine is 1960.7 h, and the frost-free period is about 247 days. The total precipitation, the mean air temperature, and the mean ground temperature during the rice season are 405 mm, 26.7 °C, and 27.8 °C in 2018, respectively (Figure 1).

The depth of the soil collected in the experiment was 0–20 cm. The soil texture was clay loam, with soil properties as follows: pH 6.9, soil total carbon 21.92 g kg^−1^, total N 1.87 g kg^−1^, total P 0.62 g kg^−1^, total K 11.94 g kg^−1^, available N 161.67 mg kg^−1^, available P 20.98 mg kg^−1^, available K 156.37 mg kg^−1^, and soil moisture content 43.33%.

Rice seedlings were transplanted into the field in June and harvested in November. Rice growth stages can be divided into the following stages: pre-transplantation, regreening, tillering, jointing, booting, heading, filling, and maturing.

### 2.2. Experimental Design

The experiment was implemented in the field where continuous rice cultivation has been practiced for 10 years (from 2009 to 2018). Three rice cultivation patterns were carried out in the experiment:

(1) Conventional farming (CF): Rice cultivation pattern with full application of chemical fertilizer;
(2) Green integrated rice-frog farming (GIRF): Rice-frog co-cropping and applied with 50% chemical and 50% organic fertilizer; and,
(3) Organic integrated rice-frog farming (OIRF): Rice-frog co-cropping and applied with 100% organic fertilizer.

In both GIRF and OIRF experimental fields, Chinese milk vetch (*Astragalus sinicus* L.) was seeded in the paddy fields after the rice harvest and ploughed into the soil the following May as Green manure (a basal fertilizer). The amount of nitrogen fertilizer applied to each treatment was the same at 300 kg N ha^−1^ [26,27,28]. The frogs used in the experiment was the tiger frog (*Rana rugulosa*), which has the main advantage of being large in size, strong adaptability, and large capacity of insect-catching. The frogs were released in the fields during the maturing stage and were left to self-sustain. During the rice growth stages, the water management was as follows: flood water layer of 5–8 cm in depth at the early stage, alternate long-term wetting state at mid-season, and field drainage at ten days before harvest (November 4th–14th). Other paddy management measures followed local high-yield field recommendations. Table 1 lists the schedule of nitrogenous fertilization for each treatment [25]. The major agricultural management practices used for the different growth stages of rice (*Oryza sativa* L.) are shown in Table 2 [25].

### 2.3. Sampling and Determination

For this study, soil parameters included TOC, C:N ratio, and soil temperature (T), while the water variables included DO, Eh, pH level, electrical conductivity (Ec), and the water level height of rice field. The DO, Eh, Ec, pH were determined by a multiparameter water quality analyzer (DZS-718L). Soil temperature was measured by a digital thermometer (TP101). The water level height was measured with a graduated scale. TOC was determined by the method of potassium dichromate oxidation. Total N was determined by a Smartchem 200 Discrete Auto Analyzer (Alliance Company, Paris, France). Rice yields per unit area were determined by theoretical method. Rice yields were the product of effective panicles per unit area, average grain number per panicle, and weight of each grain. The panicle number, grain number, and grain weight of rice were counted and weighed manually.

Field sampling was carried out manually during the 2018 rice growing season. The CH_4_ and N_2_O fluxes were simultaneously measured using the static chamber-gas chromatography (GC) method [29,30]. The sample collection chamber was made from acrylic plexiglass (7 cm thick) material and consisted of a collar, top box, and elevator box. In the center of each experimental plot, a collar with an area of 0.25 m^2^ (50 × 50 cm) was permanently inserted in the soil to a depth of 10 cm and maintained in situ over the entire rice growth period. The rice planting density in the steel chamber-base collar was consistent with the experimental plot in accordance with the common practice of local farmers [2]. The top edge of the collar had a groove (6 cm in depth) for filling with water to seal the rim of the chamber. The dimension of the top box, which was sealed at the head, was 50 × 50 × 50 cm, and each top box was equipped with two circulating fans to ensure complete gas mixing. Two holes were cut at the top and middle parts of the top box to determine the temperature and collect gas samples inside the box. The dimensions of the elevator box were the same as those of the top box. It had two covers at both ends that were allowed to open, and a groove at the top to be filled with water to seal the chamber’s rim. The elevator box was only used after rice jointing. The sample collection chamber was wrapped with a layer of aluminum foil to minimize air temperature changes inside the chamber during the period of gas sampling [31]. Gas samples were collected once a week from the 10th day after transplanting. Gas samples were taken from 08:00 to 10:00 am to closely resemble the daily average soil temperature and minimize the influence of diurnal variation in CH_4_ and N_2_O emissions during the sampling period [32]. Gas samples were collected from each chamber and were placed in pre-evacuated vacuum aluminum film airbags (produced by Dalian Delin Gas Packing Limited Company, Dalian, China) with a capacity of 100 mL at 10 min intervals (0, 10, 20, 30 and 40 min after chamber closure). The exact time of each sampling and the sample number were also recorded [33]. Gas samples in the vacuum air bags were immediately transported to the laboratory for analysis by GC within five days.

The concentrations of CH_4_ and N_2_O in the gas samples were determined using a modified GC (Agilent 7890N, Santa Clara, CA, USA), equipped with a flame ionization detector or CH_4_ analysis and an electron capture detector for N_2_O analysis [34]. The carrier gases used to carry CH_4_ and N_2_O were pure nitrogen (99.99%) and a gas mixture of argon and CH_4_, respectively. The oven and the flame ionization detector were operated at temperatures of 55 °C and 200 °C, respectively. Further details on the principles, techniques, instrument configurations, and operation procedures are discussed by Zheng et al. [35] and Wang et al. [36]. The standard gas was supplied by the National Standard Material Center. In the study, five standard gas samples were utilized to ensure the stability of the gas chromatography. Fluxes were determined based on the slope of the change in mixing ratio for five sequential samples. Sample sets were rejected unless they yielded a linear regression value of r^2^ > 0.90 [32]. The GHG emissions flux was calculated by the differences in gas concentrations in accordance with the following equation [27,28]:

F = ρ·H·dC/dt·273/ (273 + T)(1)

where F in Equation (1) represents the CH_4_ and N_2_O fluxes (mg m^−2^ h^−1^); ρ represents the CH_4_ and N_2_O density at the standard state (kg m^−3^), CH_4_ was 0.714 kg m^−3^, N_2_O was 1.964 kg m^−3^, H is the height of the chamber above the soil/water surface (m); dC/dt is the rate of change in the CH_4_ and N_2_O concentration with respect to time (t) in the chamber (mL m^−3^ h^−1^); and, T is the average air temperature inside the chamber during sampling (°C).

### 2.4. GWP and GHGI Evaluation

GWP is an index used to evaluate the combined radiative forcing potential of all the GHG, including CO_2_, CH_4_, and N_2_O. In this study, GWP expressed in CO_2_-equivalents (CO_2_-eq) was estimated, taking into account cumulative soil emissions of CH_4_ and N_2_O, assuming a 100-year time horizon [8]. The net change in TOC was not measured for this short-term experiment. The CO_2_ emissions collected by the dark chamber were not the net flux of the ecosystem, so we were not able to quantify the net CO_2_ emissions from the soils [26]. The net effects of CH_4_ and N_2_O emissions were expressed in CO_2_-equivalents according to the GWP factors provided by the Intergovernmental Panel on Climate Change (IPCC). The GWP of CH_4_ and N_2_O emissions (i.e., CO_2_ equivalents [CO_2_-eq]) were calculated using the latest GWP values: 28 for CH_4,_ and 265 for N_2_O over a 100-year time horizon [8]. The GWP was calculated through the following equation:

GWP = CH_4_ × 28 + N_2_O × 265 (2)

where GWP in Equation (2) is the global warming potential for CH_4_ and N_2_O (kg CO_2_-eq ha^−1^); and, CH_4_ and N_2_O are the total CH_4_ and N_2_O emissions (kg ha^−1^).

The GHGI is the ratio of total warming potential of CH_4_ and N_2_O to crop yield [37,38,39], such that:

GHGI = GWP/Y(3)

where GWP in Equation (3) is the total warming potential of CH_4_ and N_2_O (CO_2_ kg ha^−1^); and, Y is the average yield per unit area of the treatment (kg ha^−1^).

### 2.5. Statistical Analysis

All statistical analyses were mainly performed using the SPSS software (version 24.0, IBM, Armonk, NY, USA) and statistical significance was determined at the 0.05 probability level. Origin (version 9.1, OriginLab Co., Northampton, MA, USA) was employed for figure preparation. Differences in CH_4_ and N_2_O emissions between treatments were examined using a one-way analysis of variance (ANOVA). Correlation coefficient visibility graph was generated using the ‘ggcorrplot’ package in software R (version 3.4.2). Redundancy analysis (RDA) was performed to summarize the GHG emissions, which may be explained by the soil and water variables in paddy fields using CANOCO 5.0 software (Microcomputer Power, Ithaca, NY, USA). Finally, the fitting of the structural equation model was accomplished using the Amos 21.0 software (IBM, Armonk, NY, USA). The structural equation model is a multivariate statistical method which can describe the relationship between variables that cannot be directly measured and belongs to the confirmatory model [40].

## 3. Results

### 3.1. Environmental Factors in Rice Fields

**Figure 2:**
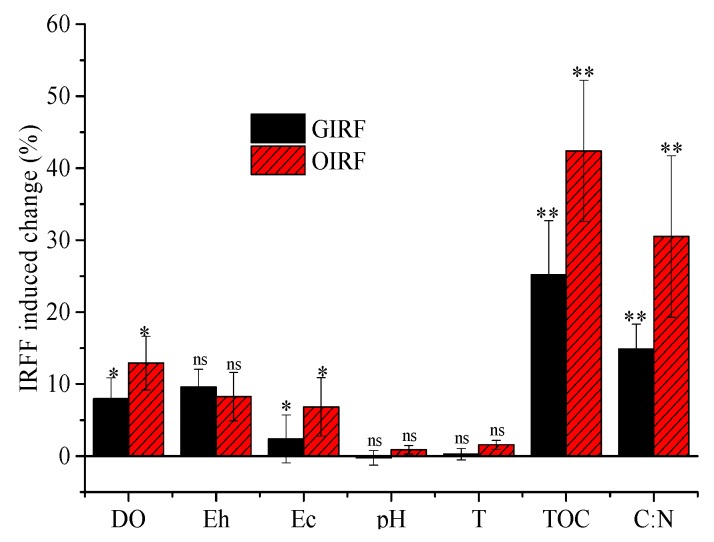
Effects of IRFF (Integrated rice-frog farming) on soil parameters during the rice growing seasons. * and ** indicate statistical significance at the 0.05 and 0.01 levels, respectively, while ns means not significant. The vertical bars indicate the standard deviation of the means (*n* = 3 replicates). GIRF: green integrated rice-frog farming; OIRF: organic integrated rice-frog farming.

Statistical analysis reveals that IRFF significantly increased the DO, soil Eh, TOC content, and soil C:N ratio in rice fields. However, the effect of IRFF on soil pH and soil temperature was minimal. In general, the effect of OIRF on soil environment of paddy fields was greater than that of GIRF. Throughout the rice planting season, IRFF significantly increased the DO, soil Eh, TOC content, and soil C:N ratio by 7.95–12.92%, 8.27–9.57%, 25.18–42.39% and 14.86–30.51%, respectively, when compared to CF (Figure 2). 

**Figure 3:**
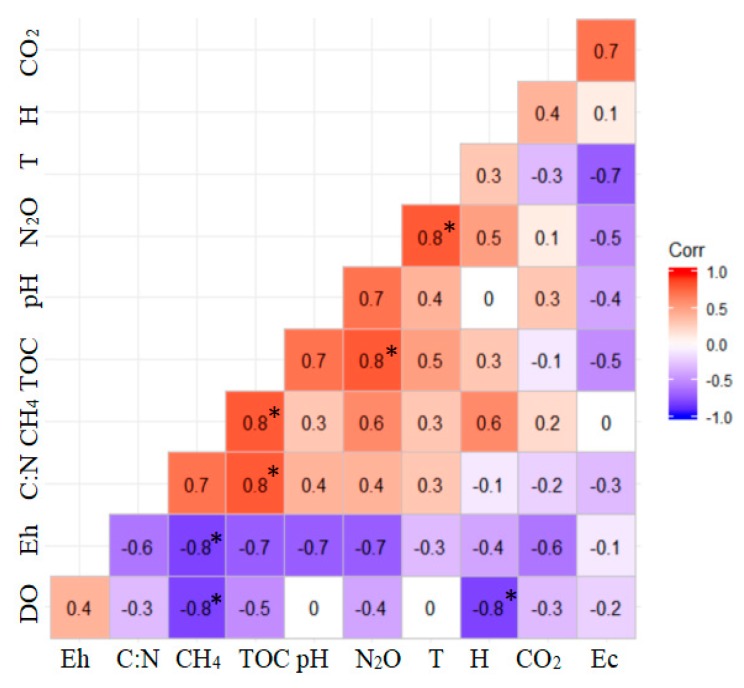
Pearson correlation coefficients for GHG (greenhouse gas) emissions against soil/water index. * indicate statistical significance at the 0.05 level.

Using Pearson correlation analysis, CH_4_ emissions were shown to be negatively correlated with TOC, DO and soil Eh, and were positively correlated with soil C:N ratio and the water level height of rice fields. The N_2_O emissions were positively correlated with TOC and soil temperature and were negatively correlated with soil Eh and Ec. The net CO_2_ emissions were significantly affected by soil Ec, Eh, water height, and pH, while TOC and C:N ratio had negligible effect (Figure 3).

### 3.2. CH_4_ Emissions

**Figure 4:**
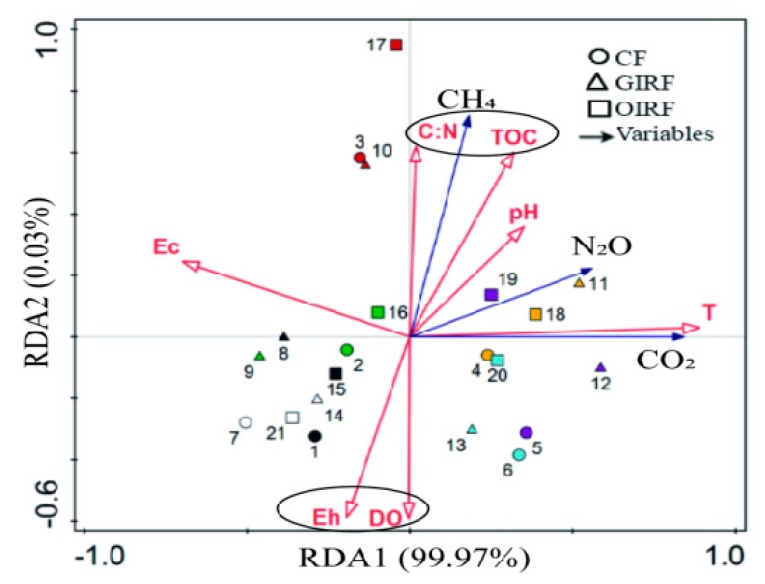
Redundancy analysis (RDA) of the relationship between the GHG emissions and soil/water variables in paddy field. Circle shape means CF treatment, triangles means GIRF, and square means OIRF. Hollow arrowheads represent environmental variables; solid arrowheads represent GHG indicators. Sample number 1 to 7 with CF treatment means regreening, tillering, jointing, booting, heading, filling, and maturing stages of rice growth, respectively. The sample number 8 to 14 of GIRF treatment are the same as CF treatment, and sample number 15 to 21 of OIRF treatment also the same as the above. Environmental factors included TOC, soil C:N ratio, DO, Eh, Ec, and soil pH. Data of soil variables are shown in the support material. Species included CH_4_, N_2_O, and CO_2_.

The relationships between the CH_4_, N_2_O, CO_2_ emissions, and soil parameters were assessed using redundancy analysis (RDA) (Figure 4). The first axis explains 99.97% of the total variation in the GHG emissions. The samples from chemical fertilizer treatment are in the third and fourth quadrants, which show that DO and Eh were significantly and negatively correlated with CF. Sample 3 shows that C:N had significant effect on CF. The GIRF data points appear scattered, the DO and Eh were negatively correlated with GIRF, while C:N, TOC, and T were positively correlated. Sample 17 shows that C:N and TOC were the main factors affecting CH_4_ emissions in totally organic fertilizer treatment of rice.

**Figure 5:**
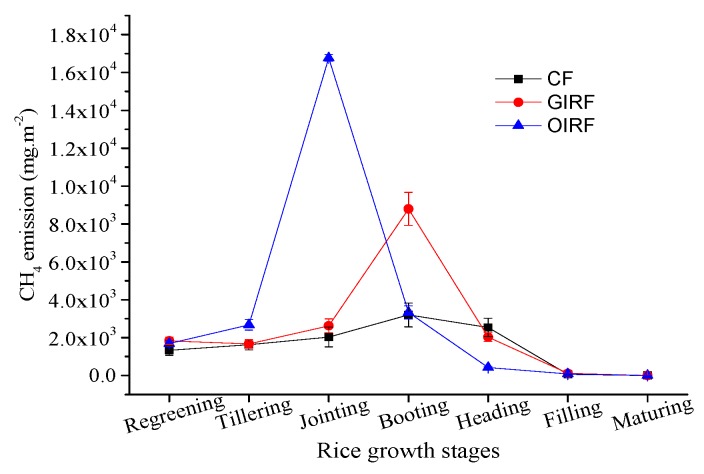
CH_4_ emissions from different growth stages of rice (*Oryza sativa* L.).

In the different stages in rice growth, the CH_4_ emissions fluxes from the three systems were different (Figure 5). The cumulative CH_4_ emissions from the entire growth period were also very different, with OIRF being the highest in value and CF being the lowest. At the rice regreening stage, the emissions of CH_4_ was lower, with values ranging from 1500 to 2000 mg m^−2^. There was no significant difference between the different systems. At the rice tillering stage, the CH_4_ emissions were more than the previous phase. The emissions of CH_4_ in OIRF were higher than CF and GIRF, possibly due to having lower DO and higher TOC in paddy waters treated with OIRF (Figure 4). The soil environment of rice fields in OIRF was more favorable in increasing the activity of methanogenic bacteria and in decreasing the activity of CH_4_-oxidizing bacteria. Thus, the amount of CH_4_ produced by OIRF is higher than that by CF and GIRF. At the rice jointing stage, CH_4_ emissions from OIRF treatment increased rapidly, reaching its peak eight and six times higher than those of CF and GIRF, respectively. The sudden increase in CH_4_ emissions from OIRF treatment was mainly due to the application of organic fertilizer (rapeseed cake) during rice jointing stage. Rapeseed cake contains a large amount of organic carbon, which provides an abundant precursor for the production of CH_4_. Another important reason is that the low content of DO in rice fields also promotes the growth of CH_4_-producing bacteria.

At the rice booting stage, CH_4_ emissions also decreased significantly due to a reduction in TOC content and an increase in DO content. The CH_4_ emissions of GIRF reached its peak at the booting stage, which may be due to sizable seepage of stored CH_4_ as a result of frog movement. During the rice heading stage, CH_4_ emissions from the different systems all showed a decreasing trend. Among them, the declines in CF and GIRF were relatively rapid. However, the CH_4_ emissions of CF and GIRF were still higher than OIRF, which may be due to the application of urea. At the rice filling stage, rice growth has almost ceased, and CH_4_ emissions were at very low levels. During the rice maturing stage, the CH_4_ emissions in all systems were very low, mainly caused by lower DO content and Eh. Although the TOC content had also been very low at this stage, the main factor was DO. Concomitantly, the water drainage in paddy fields resulted in the enhancement of soil aeration, reducing the activity of methanogenic bacteria while enhancing CH_4_-oxidizing bacteria.

The DO and Eh were significantly and negatively correlated with CF while C:N was significantly and positively correlated with GIRF. The TOC was significantly and positively related to OIRF. The C:N and TOC had significant positive correlation with CH_4_ emissions, while DO and Eh had negative effect [41,42]. Also, Ec and T did not exhibit significant correlation with CH_4_ emissions (Figure 4). Based on the entire rice growth cycle, the order of CH_4_ emissions was OIRF > GIRF > CF, and they have significant differences. This indicates that the pattern of mixed fertilization, rather than the full application of organic fertilizer, can alleviate CH_4_ emissions from paddy fields.

### 3.3. N_2_O Emissions

The pH and T had significant positive correlation with N_2_O emissions. TOC was also positively associated with N_2_O emissions, while the correlation between C:N and N_2_O emissions was very weak. Moreover, Ec, Eh, and DO were negatively associated with N_2_O emissions (Figure 4). Calculating for the whole growth period of rice, the order of N_2_O emissions was CF > GIRF > OIRF, and they have significant differences.

**Figure 6:**
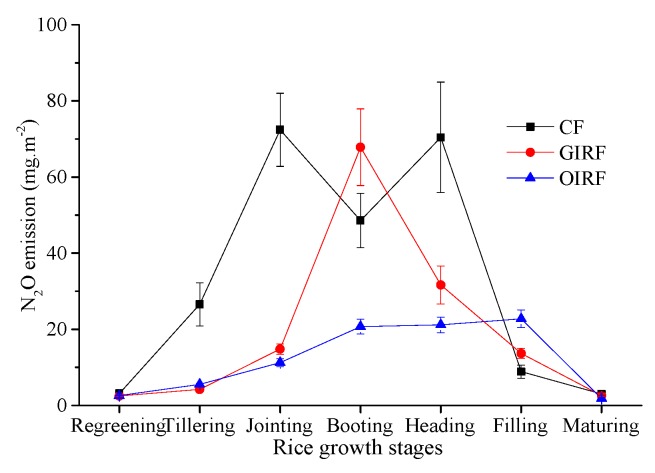
N_2_O emissions from different growth stages of rice.

In the different rice growth stages, the N_2_O emissions fluxes of the three systems were different (Figure 6). The cumulative N_2_O emissions from the entire growth period were likewise dissimilar with the order of discharge being CF > GIRF > OIRF, antithetical to CH_4_ emissions. At the rice regreening stage, the emissions of N_2_O was lower, with values below 5 mg m^−2^. This was mainly due to the small biomass of rice. At the rice tillering stage, N_2_O emissions of CF was more than GIRF and OIRF. There was also no significant difference between the two treatments. At the rice jointing stage, N_2_O emissions from CF were significantly higher than those of the other two treatments. One possible reason is that the lower C:N ratio indicates more N in the soil, providing the substrate for nitrification. In addition, less DO in the soil results in enhanced nitrification and promotes N_2_O emissions. At the rice booting stage, N_2_O emissions from CF decreased slightly, the GIRF reached its peak, and N_2_O emissions from OIRF also increased slightly. This may be due to a decrease in N content of CF, while N in both GIRF and OIRF are increased with GIRF having higher N content. Additionally, the frog activity in GIRF and OIRF have increased the DO in the soil, which further promoted the emissions of N_2_O. At the rice heading stage, the N_2_O emissions of CF was relatively high, the GIRF was in decline, and the change in OIRF was not significant. The reason was that in the CF treatment, a large amount of urea had been applied with high N content, resulting in more substrate substances producing N_2_O. At the rice filling stage, the N_2_O emissions in all three treatments were decreased, probably because the rice did not grow nutritionally and the N content in the soil decreased significantly. During the rice maturing stage, the N_2_O emissions in all treatments was very low.

### 3.4. CO_2_ Emissions

The biomass of rice was measured in the different growth stages by the method of sample plot harvesting. The CO_2_ absorbed by rice ecosystem during biomass production was then calculated using the photosynthesis Equation (4). Plants can absorb 264 g CO_2_ if they grow 162 g polysaccharide organic matter. That is, the plant can absorb 1.63 g CO_2_ for every 1 g of dry matter accumulated by the plant [43]:

6n CO_2_ + 6n H_2_O → n C_6_H_12_O_6_ + 6n O_2_ → n C_6_H_10_O_5_ 264    108    180   192    162(4)

The emissions of CO_2_ measured in field experiments were a comprehensive flux including photosynthetic fixation, rice respiration, and soil respiration in rice. The index can fully reflect the regulating function of rice ecosystem in the atmosphere, and it shows some negative values. Therefore, it is referred to as the CO_2_ absorption flux in paddy fields [43].

**Figure 7:**
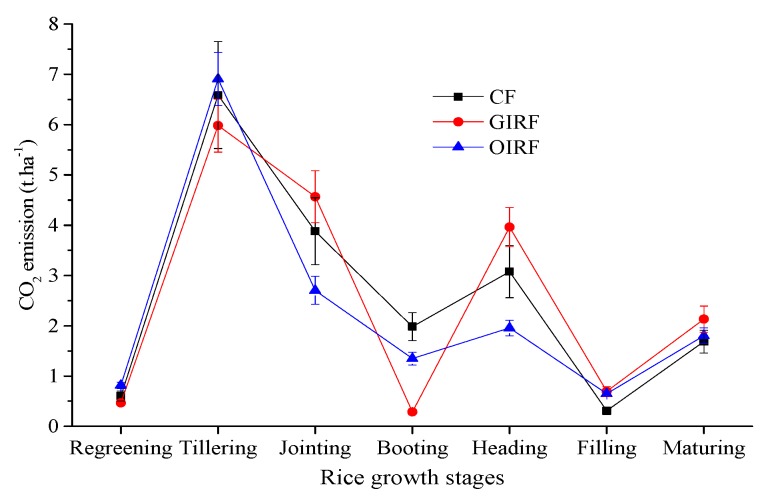
CO_2_ emissions from different growth stages of rice.

The soil temperature was significantly and positively related to the emissions of CO_2_ (Figure 4). At the tillering stage of rice, CO_2_ emissions reached its peak and then decreased. There was no significant difference among the systems in terms of net cumulative CO_2_ emissions. Generally, the average CO_2_ emissions from soils during this rice growing period were high and ranged from 2312.27 to 2589.62 kg ha^−1^ (Figure 7).

### 3.5. Rice Yield and GHGI

No growth impairment of rice plants was observed during the cropping period. Rice growth and yield properties were not significantly improved by the GIRF and OIRF systems at rice harvesting stage (Table 3). Rice yields in the GIRF and OIRF were lower (2.0% and 16.7%) than the control. The ripened grain and rice bulk density of the yield in the two IRFF systems were higher than the control. However, the other yield component was reduced by the IRFF. In comparison, rice growth and yield characteristics were not significantly different between OIRF and GIRF treatments (Table 3). Rice yield was slightly higher (17.7%) in GIRF than the OIRF treatment, but other yield properties and growth characteristics were not significantly different between the two IRFF treatments.

**Figure 8:**
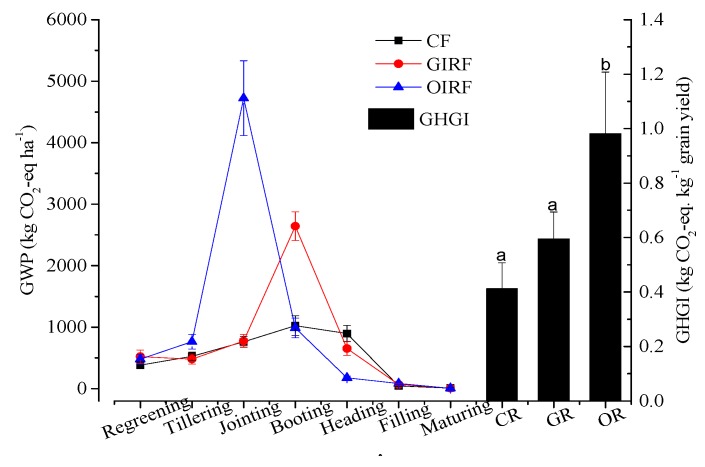
GWP (global warming potential) and GHGI (greenhouse gas intensity) during rice cultivation periods (different letters denote significant differences at *p* < 0.05 level). Vertical bars indicate standard deviations (*n* = 3 replicates).

In this study, CH_4_ emissions contributed to 83.0–96.8% of GWP; thus, the effect of IRFF on GWP was similar to CH_4_ emissions. During the rice growing seasons, IRFF significantly affected the GWP (Figure 8). Compared to CF, the treatment of GIRF and OIRF in the rice growing cycle increased the GWP by 41.3% and 98.2%, respectively. The GWP value of OIRF treatment reached its apex of 4723.63 kg ha^−1^ at the jointing stage, and subsequently decreased. For the GIRF system, the GWP value reached its peak at the booting stage (2642.57 kg ha^−1^), and then decreased gradually. The GWP value of the control treatment fluctuated slightly in the different rice growth stages. This result indicates that effective measures adopted during rice jointing and booting stages would be beneficial in mitigating GWP from paddy fields.

GHGI, which indicates net GWP per yield, was approximately 0.41 kg CO_2_-eq kg^−1^ grain in the control treatment (Figure 8). IRFF significantly increased GHGI (0.79 kg CO_2_-eq ha^−1^ grain yield), by 91.1% over the control. Compared to the complete bio-organic fertilizer application, the bulk blending fertilizer treatment decreased the GHGI by approximately 39.4% (0.59 kg CO_2_-eq ha^−1^ grain yield), which was 44.2% higher than the control.

### 3.6. Structural Equation Modeling

This study is not based only on a single factor treatment, but on the rice ecological integrated production pattern that is now being widely promoted. It is therefore uncertain how much of the results are caused by the introduction of frogs and by fertilization. Only by isolating the distinct roles of frogs and fertilization can we fully explain the impact of integrated rice-frog farming in GHG emissions. Since there are too many CO_2_-influencing factors and with the involvement of photosynthesis in rice growth, explaining the emissions mechanism clearly is more complex. And since the contributions of CH_4_ to GWP is much greater than that of N_2_O in paddy fields, this study mainly focuses on CH_4_ emissions. Using structural equation modeling, the contribution coefficient of complex environmental factors to CH_4_ emissions can be estimated.

#### 3.6.1. The Establishment of Conceptual Modeling

**Figure 9:**
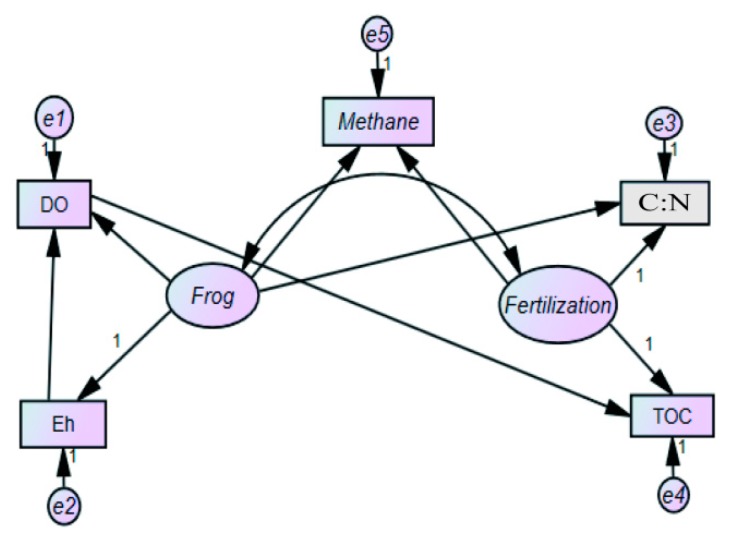
Conceptual model of CH_4_ emissions (measurement model and structural model).

Based on the characteristics of CH_4_ emissions from paddy fields, a conceptual model of the primary factors influencing CH_4_ in paddy fields was established (Figure 9). The model consists of two latent variables (Frog (ξ1) and Fertilization (ξ2)), and five measurable variables (DO (x1), Eh (x2), C:N (x3), TOC (x4), and Methane (y1). Based on the previous analysis of the primary factors, the following hypotheses are given for the conceptual model:

**Hypothesis** **1.** *Frogs have a positive effect on CH_4_ emissions from paddy field.*

**Hypothesis** **2.** *Fertilization has a positive effect on CH_4_ emissions from paddy field.*

**Hypothesis** **3.** *Correlation exists between frogs and fertilization.*

**Hypothesis** **4.** *Effects of frogs are exhibited mainly through DO, Eh, C:N and TOC.*

**Hypothesis** **5.** *Effects of fertilization are exhibited mainly through C:N and TOC.*

**Hypothesis** **6.** *Eh have an impact on DO, and DO has an impact on TOC.*

#### 3.6.2. Model Fitting Index Analysis

**Figure 10:**
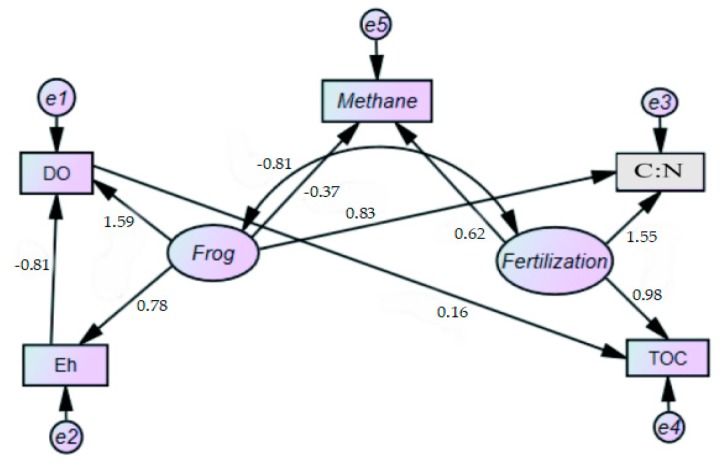
Standardized coefficients correction model for main driving factors of CH_4_ emissions from paddy fields.

For the established conceptual models and assumptions, the first initial model was fitted by using Amos 21.0. After repeated fitting, evaluation, and correction of the model, the final normalized coefficient correction model was obtained (Figure 10). By analyzing the structural equation model with Amos 21.0, better fitting indices can be obtained (Table 4). Based on previous recommendations on the structural equation model, the absolute fitting index, the relative fitting index, and the reduced index had been used in this study. The index includes CMIN/DF, GFI, RMSEA, NFI, TLI, CFI, CLE, AIC, and ECVI. The fitting standard was to be determined. The value of CMIN/DF greater than ten indicates that the model is not ideal; less than five, the model is acceptable, but a value below three would be recommended. The GFI, NFI, TLI, CFI, and IFI values should all be more than 0.9; and the closer the value is to one, the better the effect. Also, the smaller the values of AIC and ECVI, the better the fitting effect [44].

The results of the model fitting index analysis are shown in Table 4. The fitting index of the model was generally acceptable, meeting all fitting index requirements. The relationship model of CH_4_ emissions in paddy fields obtained from the statistical method was reasonable. The data in the diagram are impacted path coefficients of the modification model for balanced relations between key impact factors (Figure 10).

#### 3.6.3. Model Result Analysis

Fertilization had a positive effect on CH_4_ emissions from paddy fields, and the path coefficient was 0.62, indicating it has significant impact in increasing gas discharge (Figure 10). Frogs had a negative effect on CH_4_ emissions from paddy fields. The path coefficient was only −0.37, which was less than fertilization. CH_4_ emissions was mainly related to soil fertilization, such that the use and application of fertilizer significantly promotes CH_4_ emissions. Frog activities in paddy fields can inhibit the emissions of CH_4_, although their effects was shown to be small. There was a negative correlation between fertilization and frog behavior; the correlation coefficient was −0.81, which was consistent with the direct negative effect of frog behavior on CH_4_ emissions (Figure 10). Therefore, CH_4_ emissions from paddy fields can be reduced by modifying the quantity and mode of fertilizer used in the fields.

From the three measurable variables indicative of frog behavior, DO had the strongest contribution to frog behavior, followed by C:N, and then Eh. The path coefficients were 1.59, 0.83, and 0.78, respectively. It is widely recognized that DO has a great effect on soil CH_4_ emissions, and it is a measurable variable that affects the relation and trend. The C:N affects the content of methanogenic substrate and is also a measurable variable which contributes greatly to CH_4_ production. The influence of Eh on soil CH_4_ emissions is theoretically significant; however, Eh is highly influenced by various external factors such as solution temperature, pH, and chemical reaction reversibility. In paddy waters, the complex redox system is formed. Between the two measurable variables of the fertilization-latent variable, C:N had higher contribution to fertilizer application than the TOC. The path coefficients were 1.55 and 0.98, respectively, which indicate that C, N, and TOC have great influence on CH_4_ emissions. CH_4_ emissions from paddy field is a complex dynamic process, resulting from the interaction between the latent-to-latent variables, latent-to-measurable variables, and measurable-to-measurable variables.

In this study, both OIRF and GIRF systems were assessed on the impact of two principal factors: fertilizer use and introduction of frogs. In both systems, fertilizer and frogs were shown to play major roles in affecting the rate of CH_4_ emissions. The use of the structural equation model provides the means to further understand this complex relationship. Thus, the specific contribution of fertilizer and frog behavior to CH_4_ emissions were calculated. The results show that the contribution of fertilization to CH_4_ emissions in paddy fields is much greater than that of frog activity (Figure 10).

## 4. Discussion

### 4.1. Effects of Frogs on CH_4_ Emissions

“Frog behavior” in this study refers to the activities of frogs in rice fields, including their movement and excretion behaviors. The movement behavior of frogs can disturb the water layer and affect the DO and Eh values in paddy fields. The excretion behavior of frogs primarily involves frog feces production, which affect the TOC and C:N values in rice fields. Thus, DO, Eh, TOC, and C:N become proxy indicators for frog behavior. By using the structural equation model, we have proven that frogs’ behavior can reduce CH_4_ emissions.

The deeper question now is how do frogs reduce CH_4_ emissions? The behavior of frogs in paddy fields is similar to that of ducks, fish, and shrimp. Studies have shown that ducks and fish can affect GHG emissions, control weeds and pests, and minimize diseases in rice fields. The GWP of GHG from integrated rice-duck farming system had been studied by Yuan et al., [45] Xu et al., [46] and Zhan et al., [47]. Their results indicate that while the introduction of ducks in paddy fields can promote N_2_O emissions generated from duck feces, it also increases the concentration of DO in the water layer and reduces CH_4_ emissions. Overall, their studies found that the integrated rice-duck farming system decreases the GWP in rice fields. However, Frei et al., [48] Datta et al., [49] and Bhattacharyya et al. [23] concluded that carp production in paddy fields promotes CH_4_ diffusion and discharge through the water layer. Fish consume the DO in the water and reduced the Eh, thus increasing CH_4_ emissions. As for the introduction of shrimp, previous research have confirmed that the activities of rice shrimp could greatly increase the oxygen content in the soil and water surface [50].

In this study, our statistical analysis indicate that IRFF had significantly increased the content of DO, Eh, TOC and C:N in rice fields. The increase in DO and Eh that helps reduce CH_4_ emissions was much higher than the increases in TOC and C:N which promote greater CH_4_ emissions. Taken aggregately, the introduction of frogs provides an inhibitory effect on CH_4_ emissions in rice fields. However, this study did not dwell in analyzing frog activities in detail. We recommend that future studies investigate specific frog behavior in paddy fields to further explain the effects of frogs on CH_4_ emissions more scientifically.

### 4.2. Uncertainty and Prospect

The main limitation of the study is its use of low-frequency measurement in only one rice growing cycle. The soil environment was relatively stable, while we observed unstable meteorological factors frequently. Although we only monitored one rice growing season, we measured many indicators closely related to CH_4_ and N_2_O emissions, such as DO, Eh, TOC, soil temperature, air temperature, and water depth of paddy fields. We made sure that we recorded and observed the uncertainty factors affecting GHG emissions, reduced the random factors to a minimum, and increased the reliability and objectivity of the results. It should be noted that CH_4_ and N_2_O from paddy soil have a certain level of variability due to the variations in soil attributes and other environmental parameters. The results of GHG emissions may have been influenced by site-specific conditions, and that other locations may not generate similar results. When referring to the results of the study, the local natural meteorological parameters, soil conditions and DO, Eh, pH, TOC during sampling and monitoring should be taken into consideration, as well as the status of instruments for measuring GHG.

## 5. Conclusions

The field experiment has shown that GIRF and OIRF increased the GWP by 41.3% and 98.2% respectively for the entire rice growing period. In IRFF (included GIRF and OIRF), CH_4_ emissions from rice fields were mainly related to field fertilization, where fertilizer application can significantly promote CH_4_ emissions. Although the activity of frogs in paddy fields can inhibit the emissions of CH_4_, their effect was small. Compared to conventional farming, the introduction of frogs into the rice farming system can reduce CH_4_ emissions. The result was primarily attributed to the positive effect of frogs’ bioturbation on DO and Eh in the water layer. As a whole, although employing GIRF in rice farming showed a slight increase in GHG emissions, it could be considered as a good strategy in paddy fields for improving the agro-ecological environment and maintaining crop yield. Based on this study, the future plan is to (1) carry out more frog experiments and fertilizer experiments, and select the combination mode with the lowest GWP and better ecological benefits. (2) Then converting ecological benefits into economic benefits by adopting afforestation costs and carbon taxes, and select the experimental combination of best net ecosystem economic benefits. 

## Figures and Tables

**Table 1:** Nitrogenous fertilization scheme for each treatment.

Treatments	Pre-Transplanting			Jointing Stage		Heading Stage
	Chinese Milk Vetch	Rapeseed Cake	Bulk Blending Fertilizer	Bio-Organic Fertilizer	Bulk Blending Fertilizer	Urea
CF	None	None	150	None	75	75
GIRF	22.5	127.5	None	None	75	75
OIRF	22.5	127.5	None	150	None	None

Notes: The unit for the fertilizer application rate is kg N ha^−1^. The specific fertilization schemes of Chinese milk vetch, rapeseed cake, bulk blending fertilizer, bio-organic fertilizer, and urea were calculated from their total N contents of 0.5%, 5.3%, 26%, 6.2%, and 46%, respectively. No indication that fertilizer was not applied. Table 1 adapted from [25].

**Table 2:** Timeline and major farming management practices in experimental sites.

Rice Growth Stages	Dates	Days	Major Farming Management Practices
Pre-transplanting	14 May–14 June	31	Chinese milk vetch was ploughed in GIRF and OIRF fields (14 May), rapeseed cake was applied 100% in GIRF and OIRF fields, and bulk blending fertilizer was applied 67% in CF fields (14 June).
Regreening	15 June–30 June	15	
Tillering	1 July–22 July	21	Tiger frogs were put into GIRF and OIRF fields (4500 and 6000 frogs ha^−1^ on 1 July). Bulk blending fertilizer were applied 33% in CF and 100% in GIRF, while 100% bio-organic fertilizer in OIRF fields (15 July).
Jointing	23 July–12 August	20	Weed removal
Booting	13 August–31 August	18	Urea (100%) was applied in CF and GIRF fields (15 August).
Heading	1 September–18 September	17	Irrigating water
Filling	19 September–9 October	20	Weed removal
Maturing	10 October–29 October	19	Chinese milk vetch was sowed in GIRF and OIRF fields (20 October)
Harvesting	14 November	1	Rice harvested on 14 November

Note: Different field management measures were used in different growing stages of rice. The total number of rice growth days were 131 days. The management of the field was carried out in accordance with the local high-yield pattern. Table 2 adapted from [25].

**Table 3:** Rice growth and yield properties at rice harvesting stage.

Parameters	CF	GIRF	OIRF
Biomass yield (kg ha^−1^)			
rice yield	8827.56 a *	8650.38 a *	7350.69 b *
Straw	30,441.6 a	31,302.0 a	26,690.4 a
Total above-ground	39,269.16 a	39,952.38 a	34,041.09 a
Plant height (cm)	103.5 a	100.8 a	100.2 a
Straw stem diameter (mm)	5.7 a	5.7 a	5.8 a
Tiller number per hill	13 a	13 a	12 a
Grains per panicle	86 a	88 a	85 a
Ripened grains (%)	75% a	78% a	80% a
1000 grain weight (g)	22 a	21 a	20 a
Rice bulk density (kg L^−1^)	0.52 a	0.55 a	0.57 a

* Mean values followed by different letters in the same line indicate significance difference among treatments at *p* < 0.05.

**Table 4:** Fitting coefficients list of the structural equation model.

Indices Name		Evaluation Criterion	Results
Absolute fitting index	CMIN/DF	<3	1.188
	GFI	>0.9	0.933
Relative fit index	NFI	>0.9	0.961
	TLI	>0.9	0.909
	CFI	>0.9	0.991
Compact index	IFI	>0.9	0.994
	AIC	The smaller, the better	29.188
	ECVI	The smaller, the better	4.865

## References

[1] Xia L.L., Xia Y.Q., Ma S.T., Wang J.Y., Wang S.W., Zhou W., Yan X.Y. (2016). Greenhouse gas emissions and reactive nitrogen releases from rice production with simultaneous incorporation of wheat straw and nitrogen fertilizer. Biogeosciences.

[2] Zhou M.H., Zhu B., Wang X.G., Wang Y.Q. (2017). Long-term field measurements of annual methane and nitrous oxide emissions from a Chinese subtropical wheat-rice rotation system. Soil Biol. Biochem..

[3] Jiang Y., Qian H., Wang L., Feng J.F., Huang S., Hungate B.A., Kessel C.V., Horwath W.R., Zhang X.Y., Qin X.B. (2019). Limited potential of harvest index improvement to reduce methane emissions from rice paddies. Glob. Chang. Biol..

[4] Carlson K.M., Gerber J.S., Mueller N.D., Herrero M., MacDonald G.K., Brauman K.A., Havlik P., O’Connell S., Johnson J.A., Saatchi S. (2017). Greenhouse gas emissions intensity of global croplands. Nat. Clim. Chang..

[5] Liu S.W., Hu Z.Q., Wu S., Li S.Q., Li Z.F., Zou J.W. (2016). Methane and Nitrous Oxide Emissions Reduced Following Conversion of Rice Paddies to Inland Crab-Fish Aquaculture in Southeast China. Environ. Sci. Technol..

[6] Zhao X., Liu S.L., Pu C., Zhang X.Q., Xue J.F., Zhang R., Wang Y.Q., Lal R., Zhang H.L., Chen F. (2016). Methane and nitrous oxide emissions under no-till farming in China: A meta-analysis. Glob. Chang. Biol..

[7] Qin H.L., Tang Y.F., Shen J.L., Wang C., Chen C.L., Yang J., Liu Y., Chen X.B., Li Y., Hou H.J. (2018). Abundance of transcripts of functional gene reflects the inverse relationship between CH_4_ and N_2_O emissions during mid-season drainage in acidic paddy soil. Biol. Fertil. Soils.

[8] Stocker T.F., Qin D., Plattner G.K., Tignor M., Allen S.K., Boschung J., Nauels A., Xia Y., Bex V., Midgley P.M., IPCC (2013). Climate Change: The Physical Science Basis.

[9] Olesen J.E., Bindi M. (2002). Consequences of climate change for European agricultural productivity, land use and policy. Eur. J. Agron..

[10] Cohn A.S., VanWey L.K., Spera S.A., Mustard J.F. (2016). Cropping frequency and area response to climate variability can exceed yield response. Nat. Clim. Chang..

[11] Mora C., Spirandelli D., Franklin E.C., Lynham J., Kantar M.B., Miles W., Smith C.Z., Freel K., Moy J., Louis L.V. (2018). Broad threat to humanity from cumulative climate hazards intensified by greenhouse gas emissions. Nat. Clim. Chang..

[12] Feng X., Jiang C.S., Peng X.L., Li Y.P., Hao Q.J. (2019). Effects of the crop rotation on greenhouse gases from flooded paddy fields. Environ. Sci..

[13] Jeong S.T., Kim G.W., Hwang H.Y., Kim P.J., Kim S.Y. (2018). Beneficial effect of compost utilization on reducing greenhouse gas emissions in a rice cultivation system through the overall management chain. Sci. Total Environ..

[14] Haque M.M., Kim G.W., Kim P.J., Kim S.Y. (2016). Comparison of net global warming potential between continuous flooding and midseason drainage in monsoon region paddy during rice cropping. Field Crops Res..

[15] Xie J., Hu L.L., Tang J.J., Wu X., Li N.N., Yuan Y.G., Yang H.S., Zhang J.E., Luo S.M., Chen X. (2011). Ecological mechanisms underlying the sustainability of the agricultural heritage rice–fish coculture system. PNAS.

[16] Lu Y.H., Liao Y.L., Nie J., Zhou X., Fu X.Q., Huang L. (2017). Research and Prospect of Rice-Frog Ecological Cultivation and Breeding Technology Mode. Hunan Agric. Sci..

[17] Cai C., Li G., Zhu J.Q., Peng L., Li J.F., Wu Q.X. (2019). Effects of Rice-crawfish Rotation on Soil Physicochemical Properties in Jianghan Plain. Acta Pedol. Sin..

[18] Liu G.P., Zhang Y.Z., Huang Z.N., Chen K.L., Liu Y., Zhu G.Q., Fang B.H. (2013). Effects of Rice-Bullfrog Mixed Cultivation on Rice Planthoppers and Rice Yield. Chin. J. Biol. Contr..

[19] Liang X., Li H., Wang S., Ye Y., Ji Y., Tian G., Van Kessel C., Linquist B. (2013). Nitrogen management to reduce yield-scaled global warming potential in rice. Field Crops Res..

[20] Mosier A., Kroeze C. (2000). Potential impact on the global atmospheric N_2_O budget of the increased nitrogen input required to meet future global food demands. Chemosphere Glob. Chang. Sci..

[21] Xiong Z.Q., Xing G.X., Tsuruta H., Shi S.L., Shen G.Y., Du L.J. (2003). Nitrous oxide emissions from paddy soils as affected by incorporation of leguminous green manure and fertilization during double-cropping rice-growing season. Acta Pedol. Sin..

[22] Xie Y.Q., Zhang J.F., Jiang H.M., Yang J.C., Deng S.H., Li X., Guo J.M., Li L.L., Liu X., Zhou G.Y. (2015). Effects of different fertilization practices on greenhouse gas emissions from paddy soil. J. Agro-Environ. Sci..

[23] Bhattacharyya P., Sinhababu D.P., Roy K.S., Dash P.K., Sahu P.K., Dandapat R., Neogi S., Mohanty S. (2013). Effect of fish species on methane and nitrous oxide emission in relation to soil C, N pools and enzymatic activities in rainfed shallow lowland rice-fish farming system. Agric. Ecosyst. Environ..

[24] Adviento-Borbe M.A.A., Linquist B. (2016). Assessing fertilizer N placement on CH_4_ and N_2_O emissions in irrigated rice systems. Geoderma.

[25] Yi X.M., Yuan J., Zhu Y.H., Yi X.J., Zhao Q., Fang K.K., Cao L.K. (2018). Comparison of the Abundance and Community Structure of N-Cycling Bacteria in Paddy Rhizosphere Soil under Different Rice Cultivation Patterns. Int. J. Mol. Sci..

[26] Zhao Z., Yue Y.B., Sha Z.M., Li C.S., Deng J., Zhang H.L., Gao M.F., Cao L.K. (2015). Assessing impacts of alternative fertilizer management practices on both nitrogen loading and greenhouse gas emissions in rice cultivation. Atmos. Environ..

[27] Yuan J., Sha Z.M., Hassani D., Zhao Z., Cao L.K. (2017). Assessing environmental impacts of organic and inorganic fertilizer on daily and seasonal Greenhouse Gases effluxes in rice field. Atmos. Environ..

[28] Yuan J., Yuan Y.K., Zhu Y.H., Cao L.K. (2018). Effects of different fertilizers on methane emissions and methanogenic community structures in paddy rhizosphere soil. Sci. Total Environ..

[29] Zhou M.H., Zhu B., Bruggemann N., Wang X.G., Zheng X.H., Butterbach-Bahl K. (2015). Nitrous oxide and methane emissions from a subtropical rice-rapeseed rotation system in China: A 3-year field case study. Agric. Ecosyst. Environ..

[30] Wang W.Q., Sardans J., Wang C., Zeng C.S., Tong C., Asensio D., Penuelas J. (2017). Relationships between the potential production of the greenhouse gases CO_2_, CH_4_ and N_2_O and soil concentrations of C, N and P across 26 paddy fields in southeastern China. Atmos. Environ..

[31] Shang Q.Y., Yang X.X., Gao C.M., Wu P.P., Liu J.J., Xu Y.C., Shen Q.R., Zou J.W., Guo S.W. (2011). Net annual global warming potential and greenhouse gas intensity in Chinese double rice-cropping systems: A 3-year field measurement in long-term fertilizer experiments. Glob. Chang. Biol..

[32] Zou J.W., Huang Y., Jiang J.Y., Zheng X.H., Sass R.L. (2005). A 3-year field measurement of methane and nitrous oxide emissions from rice paddies in China: Effects of water regime, crop residue, and fertilizer application. Glob. Biogeochem. Cycles.

[33] Zhong J., Fu Z.Q., Liu L., Zhu Z.J., Zheng H.B. (2017). Correlation Analysis of Methane Transport Capacity and Root Characteristics in Rice. Crop.

[34] Hoang T.T.H., Do D.T., Tran T.T.G., Ho T.D., Rehman H.U. (2019). Incorporation of rice straw mitigates CH_4_ and N_2_O emissions in water saving paddy fields of Central Vietnam. Arch. Agron. Soil Sci..

[35] Zheng X., Mei B., Wang Y., Xie B., Wang Y., Dong H., Xu H., Chen G., Cai Z., Yue J. (2008). Quantification of N_2_O fluxes from soil-plant systems may be biased by the applied gas chromatograph methodology. Plant Soil.

[36] Wang Y.H., Wang Y.S., Ling H. (2010). A new carrier gas type for accurate measurement of N_2_O by GC-ECD. Adv. Atmos. Sci..

[37] Li C., Salas W., DeAngelo B., Rose S. (2006). Assessing alternative for mitigating net greenhouse gas emissions and increasing yields from rice production in China over the next twenty years. J. Environ. Qual..

[38] Mosier A.R., Halvorson A.D., Reule C.A., Liu X.J. (2006). Net global warming potential and greenhouse gas intensity in irrigated cropping systems in Northeastern Colorado. J. Environ. Qual..

[39] Qin Y., Liu S., Guo Y., Liu Q., Zou J. (2010). Methane and nitrous oxide emissions from organic and conventional rice cropping systems in Southeast China. Biol. Fertil. Soils.

[40] Hou J.T., Wen Z.L., Cheng Z.J. (2004). Structural Equation Model and Its Application.

[41] Yan X.Y., Yagi K., Akiyama H., Akimoto H. (2005). Statistical analysis of the major variables controlling methane emission from rice fields. Glob. Chang. Biol..

[42] Peters M., Conrad R. (2006). Sequential reduction processes and initiation of CI-h production upon flooding of oxic upland soils. Soil Biol. Biochem..

[43] Xiao Y., Xie G.D., Lu C.X., Ding X.Z., Lv Y. (2004). The gas regulation function of rice paddy ecosystems and its value. J. Nat. Resour..

[44] Li H., Wang J.K., Pei J.B., Li S.Y. (2015). Equilibrium relationships of soil organic carbon in the main croplands of northeast china based on structural equation modeling. Acta Ecol. Sin..

[45] Yuan W.L., Cao C.G., Li C.F., Zhan M., Cai M.L., Wang J.P. (2009). Methane and Nitrous Oxide Emissions from Rice-Fish and Rice-Duck Complex Ecosystems and the Evaluation of Their Economic Significance. Sci. Agric. Sin..

[46] Xu G.C., Liu X., Wang Q.S., Yu X.C., Hang Y.H. (2017). Integrated rice-duck farming mitigates the global warming potential in rice season. Sci. Total Environ..

[47] Zhan M., Cao C.G., Wang J.P., Li C.F., Yuan W.L. (2009). Greenhouse gas emission from an integrated rice-duck system and its global warming potentials. Acta Scientiae Circumstantiae.

[48] Frei M., Becker K. (2005). Integrated rice-fish production and methane emission under greenhouse conditions. Agric. Ecosyst. Environ..

[49] Datta A., Nayak D.R., Sinhababu D.P., Adhya T.K. (2009). Methane and nitrous oxide emissions from an integrated rainfed rice–fish farming system of Eastern India. Agric. Ecosyst. Environ..

[50] Xu X.Y., Zhang M.M., Peng C.L., Si G.H., Zhou J.X., Xie Y.Y., Yuan J.F. (2017). Effect of rice-cray fish co-culture on greenhouse gases emission in straw-puddled paddy fields. Chin. J. Eco Agric..

